# Pedunculopontine Nucleus Stimulation: Where are We Now and What Needs to be Done to Move the Field Forward?

**DOI:** 10.3389/fneur.2014.00243

**Published:** 2014-12-04

**Authors:** Hokuto Morita, Chris J. Hass, Elena Moro, Atchar Sudhyadhom, Rajeev Kumar, Michael S. Okun

**Affiliations:** ^1^Department of Neurology, University of Florida Center for Movement Disorders and Neurorestoration, Gainesville, FL, USA; ^2^Department of Applied Physiology and Kinesiology, University of Florida, Gainesville, FL, USA; ^3^Department of Psychiatry and Neurology, CHU de Grenoble, Grenoble, France; ^4^Department of Radiation Oncology, University of California at San Francisco, San Francisco, CA, USA; ^5^Rocky Mountain Movement Disorder Center, Denver, CO, USA

**Keywords:** pedunculopontine nucleus, deep brain stimulation, Parkinson’s disease, microelectrode recording, postural instability, gait freezing, diffusion tractography

## Abstract

Falls and gait impairment in Parkinson’s Disease (PD) is a leading cause of morbidity and mortality, significantly impacting quality of life and contributing heavily to disability. Thus far axial symptoms, such as postural instability and gait freezing, have been refractory to current treatment approaches and remain a critical unmet need. There has been increased excitement surrounding the surgical targeting of the pedunculopontine nucleus (PPN) for addressing axial symptoms in PD. The PPN and cuneate nucleus comprise the mesencephalic locomotor region, and electrophysiologic studies in animal models and human imaging studies have revealed a key role for the PPN in gait and postural control, underscoring a potential role for DBS surgery. Previous limited studies of PPN deep brain stimulation (DBS) in treating gait symptoms have had mixed clinical outcomes, likely reflect targeting variability and the inherent challenges of targeting a small brainstem structure that is both anatomically and neurochemically heterogeneous. Diffusion tractography shows promise for more accurate targeting and standardization of results. Due to the limited experience with PPN DBS, several unresolved questions remain about targeting and programing. At present, it is unclear if there is incremental benefit with bilateral versus unilateral targeting of PPN or whether PPN targeting should be performed as an adjunct to one of the more traditional targets. The PPN also modulates non-motor functions including REM sleep, cognition, mood, attention, arousal, and these observations will require long-term monitoring to fully characterize potential side effects and benefits. Surgical targeting of the PPN is feasible and shows promise for addressing axial symptoms in PD but may require further refinements in targeting, improved imaging, and better lead design to fully realize benefits. This review summarizes the current knowledge of PPN as a DBS target and areas that need to be addressed to advance the field.

## Current Status of PPN DBS

Major morbidity and mortality in Parkinson’s disease (PD) have been closely linked to problems arising from falling. Indeed, the resultant sequelae from hip fracture and aspiration pneumonia are both appreciated as leading causes of PD related death (1). Long-term pharmacological therapy has failed in adequately treating axial motor symptoms of PD including fall related morbidity, ambulatory difficulties, balance problems, freezing, and also swallowing dysfunction. Deep brain stimulation (DBS) addresses primarily PD motor symptoms with the most commonly utilized stimulation targets being the subthalamic nucleus (STN) and the globus pallidus internus (GPi) (2–5). Stimulation of these two specific targets, while producing striking improvements in tremor, bradykinesia, rigidity, and levodopa-induced dyskinesia (6–8) have unfortunately fallen short in alleviating important disease related PD morbidities, especially levodopa unresponsive gait impairment, freezing, falling, and swallowing issues.

Thus an unmet but pressing need exists to develop novel therapeutic strategies to treat disabling levodopa and DBS resistant axial disturbances in PD. Because the pedunculopontine nucleus (PPN) is an integral component of the mesencephalic locomotor system, and because it coexists within a rich brainstem network thought to have a modulatory role in many motor and non-motor PD features, there has been recent enthusiasm for this novel target. Indeed, early and promising data suggested that the PPN could be a viable DBS target for treating axial symptoms. Several small non-randomized, non-controlled studies examining short-term outcomes have been performed (see Table 1).

**Table 1:** **Studies on PD patients implanted with PPN DBS**.

Study	Subjects	Target	Inclusion	Study design	Effect on FOG/PI	Falls	UPDRSIII	Adverse events	Comments
Plaha and Gill (9)	2	Bilateral PPNPPN	FOG, PI, falls in both the ON and OFF states	Open label	+ (2/2) ON and OFF medication	+ (2/2) ON and OFF medication	+ (2/2) In both ON and OFF medication states	Worsened gait and motor at certain frequencies	Patients had contrasting profiles at higher frequencies, follow up at 16 and 42 days
Strafella et al. (10)	1	Unilateral PPN	Advanced PD of at least 5 years duration, > 30% benefit in ON state, FOG, PI	Open label	Benefit on gait subscore of UPDRSIII	NA	+ (1/1) OFF medication benefit of 19% on UPDRSIII	NA	PET study showing increased rCBF in subcortical areas bilaterally, thalamus
Brusa et al. (11)	1	Unilateral PPN	PSP-P w/FOG, PI	Open label	0	0	+ (1/1) UPDRSIII 22 to –18 at 3 months	Intensity dependent paresthesias	Slight improvement in subjective gait ignition failure
Moro et al. (12)	6	Unilateral PPN	Age < 70, absence of dementia, severe off PI and FOG	Double blind	0	+ (in falls at 3 and 12 months, falling score 70% improved	0 (no difference in ON and OFF stim in ON and OFF med states at 3 and 12 months)	Intensity and frequency dependent paresthesias, oscillopsia	Medication responsive on gait, posture, freezing subscores of UPDRS
Ostrem et al. (13)	1	Bilateral PPN	PPFG	Open label	+ (FOG questionnaire)	0	0	None	Mild improvement on FOG questionnaire not sustained at 12 months
Thevathasan et al. (14)	5	Bilateral PPN	Severe FOG, PI, falls persisting in ON state	Open label	+ (5/5 patients by questionnaire at 3 months and 2 years)	+ (5/5 at 6 months and 4/5 at 2 years)	0	Oscillopsia, frequency dependent worsening of motor and gait	Followed over 2 years.
Wilcox et al. (15)	1	Bilateral PPN	Primary progressive freezing of gait	Open label	+ (questionnaire and gait testing)	+ (questionnaire)	+ (1/1)	None reported	Followed to 14 months
Aviles-Olmos et al. (16)	1	Unilateral PPN	Gait and balance impairment in on state	Open label	+	0	0	Urinary incontinence	Gait analysis showed improvement in freezing and velocity but not sufficient to improve UPDRS subscores
Thevathasan et al. (17)	7	5 bilateral PPN, 2 unilateral PPN	FOG, PI, falls persisting in the on state		+ (6/6 patients at follow up 2—13 months by questionnaire, less robust in unilateral patients)	+	NA	Not reported	1 patient published in previous study, gait freezing improved OFF stim for up to 6 weeks
Stefani et al. (18)	6	Bilateral STN + PPN	UPDRSIII > 70, disabling axial symptoms, 3 of 6 had ON medication FOG	Open label	PPN had much better benefit on posture and gait items compared to STN.	NA	+ (6/6) ON and OFF PPN only ON mean 32% improvement in UPDRSIII compared to 54% improvement with STN ON.	paresthesias	Slight decline in efficacy of PPN after 6 months, trend toward increased benefit on UPDRS III with both STN and PPN ON
Androulidakis et al. (19)	6	2 bilateral STN + PPN, 2 unilateral STN + PPN, 1 unilateral GPi + PPN, 1 unilateral PPN	Severe PD, FOG	Open label	NA	NA	+ (6/6) in ON medication state	Not reported	Intraoperative recording. Clinical gait measures not reported. Unilateral PPN case previously reported in (18).
Mazzone et al. (20)	14	8 bilateral, 6 unilateral 5 bilateral PPN + STN, 1 bilateral PPN with 1 lead explanted, 5 unilateral PPN, 1 unilateral PPN + GPi, 1 bilateral GPi + CM-PF	12 PD and 2 PSP	Open label	+ (mean improvement on posture and gait subscores of UPDRS)	NA	+	Transient paresthesias	Follow up at 15 day test period for acute and 1–24 months for chronic effects of DBS.
Thevathasan et al. (21)	11	8 bilateral PPN, 2 bilateral PPN + ZI, 1 unilateral PPN + bilateral ZI	FOG, PI, falls persisting in ON state	Open label	+	+ (10/11)	0	None reported	Gait, posture, balance assessed by UPDRS subscores 27–30, follow up at 3–38 months
Ferraye et al. (22)	6	Bilateral PPN + STN	Severe gait and freezing despite STN	Double blind	0 (composite gait score, FOG questionnaire, duration of freezing)	0	0	Seizure in 1 pt, frequency dependent oscillopsia, paresthesias, limb myoclonus	Objective freezing improved in 2 patients in levodopa ON. Outcomes at baseline and 1 year follow up.
Mazzone et al. (23)	23	6 bilateral STN + PPN, 3 bilateral GPi + unilateral PPN, 1 unilateral GPi + unilateral PPN, 13 unilateral PPN	22 PD and 1 PSP	Open label	+	NA	+ (23/23)	Transient paresthesias reported in earlier series	Includes patients from Mazzone et al. (20, 24). Last 17 patients had unilateral PPN showing similar benefits to traditional targets of STN and GPi.
Khan et al. (25)	7	Bilateral PPN + ZI	FOG, PI, falls in both off and on	Open label	+ (improvement in axial subscores with both PPN and ZI on)	NA	+ (both OFF and ON medication states, ZI had greater benefit than PPN)	Self limited akinesia postop in 2 patients	No significant diff between ZI ON versus both PPN and ZI ON for UPDRSIII, follow up at baseline and 12 months, trend toward improvement with both, sig with axial subscore
Franzini et al. (26)	2	Unilateral 1 STN + PPN, 1PPN	Falls and gait difficulty, one patient had akinesia due to neuroleptic abuse	Open label	NA	NA	+ (UPDRSIII 29 to 15 in one patient, gait improved in other)	Not reported	Gait and falls improved by report but not quantified.

*PD, Parkinson disease; PPN, pedunculopontine nucleus; STN, subthalamic nucleus; GPi, globus pallidus, internal segment; ZI, Zona Incerta; FOG, freezing of gait; PI, postural instability; UPDRS, United Parkinson Disease Rating Scale; rCBF, regional cerebral blood flow; +, improvement; 0, no improvement, NA, not available*.

The excitement in the field about a novel DBS target (PPN) potentially capable of addressing medication resistant axial motor symptoms (freezing, falling, and swallowing) has been tempered by mixed clinical outcomes that have ranged from excellent to uncertain. Almost all of the studies have been open label and have not had rigidly defined inclusion criteria. Also, there has not been a standardized outcome measures battery of both subjective and objective measure of freezing of gait (FOG), postural instability, and falls. Most studies have used either the gait and posture subscores of the UPDRSIII or FOG and gait and falls questionnaires. PPN DBS studies have been summarized in Table 1.

The Plaha et al. study showed impressive improvements in UPDRS III motor scores as well as improvements with falls and FOG in both the ON and OFF medication states in two patients with bilateral STN DBS plus bilateral PPN DBS, however, the study was open label, and there was no clearly defined outcome measures for FOG, falls, and postural instability. This report was followed by Stefani and colleagues’ study who also evaluated bilateral STN and bilateral PPN DBS as a combination treatment. Stefani’s team performed a six patient open label study without rigid inclusion criteria, and like Plaha they reported impressive UPDRS III motor changes in both the ON med and OFF med states. PPN DBS improved gait and postural stability more than STN DBS, but did not improve limb function as much as STN DBS. However, the combination of PPN and STN DBS improved both axial and limb function more than either target alone. In a larger series by Mazzone et al. in a series of 14 patients that consisted of a combination of unilateral and bilateral PPN targeting, some with concurrent or previous targeting of STN, GPi, there were mean improvements of UPDRS III and posture and gait subscores of UPDRS III (20). The results of these open label studies have been mitigated to some extent by Ferraye’s recent report of a double blind trial of bilateral STN and bilateral PPN DBS in six patients. Ferraye’s group concluded that freezing and falling improved in 5/6 patients, but the UPDRS III gait items did not improve. In this study, medication status (ON versus OFF freezing) and falling were not detailed in the inclusion criteria. Moro et al. in 2010 reported that in six patients with unilateral PPN DBS (no previous STN surgery), the UPDRS motor scores did not improve with a double blind evaluation, however, falls on the UPDRS II scale were decreased (12). The Moro study did not use bilateral implants, and only required participants to exhibit falling in the off medication state, and not in the on medication state. Thevathasan and coworkers have performed a series of studies looking at patients with bilateral PPN DBS without implantation of other traditional targets. The patients in these studies all had FOG, postural instability, and falls persisting in the ON medication state as inclusion criteria and all had improvements in FOG, postural instability, and falls using questionnaire as an outcome measure, however, these patients did not have improvements in non-axial associated UPDRS measures (14). Improvements in FOG, PI, and falls seemed to be less robust in patients implanted in PPN unilaterally though the data were limited (17). The approach of the Mazzone and coworkers group has evolved with technical improvements in their procedure and increased experience, now with at least 23 patients with PPN implantations (23). Initially, their patients were implanted with both PPN and STN DBS simultaneously in one hemisphere then 10–15 days later in the contralateral PPN and STN. With refinements in their techniques, they have since reported switching to a preferred strategy of unilateral PPN targeting with consideration of PPN as a primary target (23). In this study, there were no significant differences in the UPDRS subscores 27–30 and OFF DBS and ON DBS when comparing patients with combined implantations and those with unilaterally implanted PPN.

Several critical issues must be clarified to truly determine the viability of PPN DBS for the treatment of PD related axial symptoms. First, examination of the efficacy of PPN DBS should be performed in a carefully selected drug-resistant population, and it should be performed with and without existing STN or GPi DBS systems in order to sort out its true therapeutic effects. Such an examination will clarify the independent role and potential additive benefits of PPN DBS on drug-resistant axial PD symptoms. Furthermore, this type of careful examination will aid in determining the patient characteristics that may predict a better outcome.

Additionally, optimal localization of lead placement within the PPN is not trivial. In fact, placement of DBS leads in the brainstem PPN region has produced more variability in targeting than has been previously observed for other DBS procedures. This feature, until resolved, possibly limits the PPN as a viable surgical target. More rigorous and advanced targeting and localization procedures are needed with careful follow-up imaging and likely tractography. Ultimately, this may require advancements in lead design to create PPN specific leads.

Historically, the initial studies of GPi and STN DBS dramatically underreported the incidence and nature of motor and non-motor adverse effects. There has also been significant concern about potential adverse events resulting from PPN DBS as the PPN is located in a region close to many important brainstem centers. Because there have been so few reports of PPN DBS, adverse event data have been sparse and mainly limited to oscillopsia, paresthesias, and urinary incontinence (12, 16, 18, 22, 27), which may vary depending on target location. Some of the motor side effects can be predicted based on the anatomic relationship of the PPN, however, the PPN also plays a role in modulating several non-motor systems with less predictable effects. Careful and critical examination and reporting of adverse events are needed to clarify the safety of PPN DBS and to determine which patient characteristics may place a patient at greatest risk.

Finally, the precise nature of axial motor dysfunction and incidence of falling pre- and post-PPN DBS need to be quantified. These objective and sensitive analyses will require specific and well-selected outcome measures. From these, clinical trials will be in a better position to sort out the features that are captured by PPN stimulation, and what patient characteristics or clinical outcome measures may predict a better outcome from surgery. In this paper, we will address what is known about PPN DBS, and we will make recommendations to help the field move forward.

## PPN Anatomy, Physiology, and Imaging Guidance for DBS

In advanced PD, many deficits including problems with ambulation, postural instability, FOG, falls, and swallowing disturbances are levodopa refractory, and thus not entirely explained by dysfunction of nigrostriatal dopaminergic pathways (28–32). Therefore, a likely explanation is that in parallel to the nigro-striatal-pallido-thalamo-cortical neuronal circuits mediating voluntary movements, there exist reciprocal pathways between the basal ganglia and brain stem/spinal cord nuclei that may modulate at least some of these functions [reviewed in Ref. (33)]. Recent evidence suggests that the PPN plays an integral role in deficits seen in PD, and since the control of locomotion and posture seem to depend more on the brainstem than on cortical control, attention has turned to this brainstem based nucleus, in particular the mesencephalic locomotor region (MLR) (34–36).

**Figure 1:**
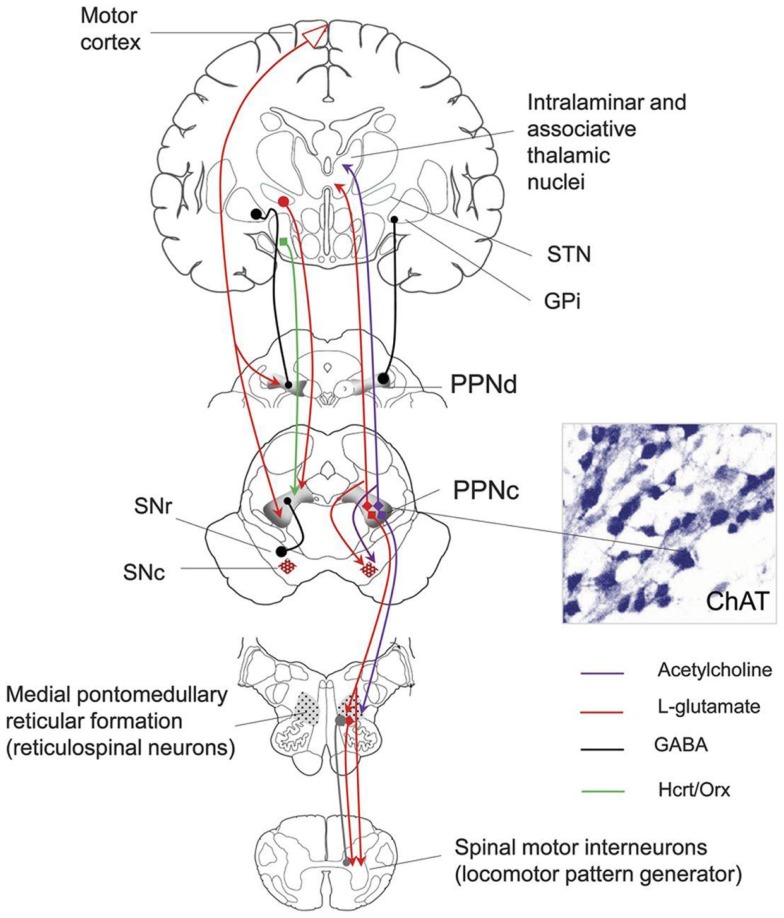
**Anatomical connections of the PPN**. SNr, substantia nigra; SNc, substantia nigra pars compacta; STN, subthalamic nucleus; GPi, globus pallidus internal segment; PPNc, pedunculopontine nucleus pars compacta; PPNd, pedunculopontine nucleus pars dissipatus; ChAT, choline acetyltransferase. Image from the American Academy of Neurology image library.

The MLR consists of the PPN and the cuneate nucleus and is both anatomically and neurochemically heterogeneous (Figure 1) consisting of cholinergic, non-cholinergic, and mixed neurons (37).

In normal humans, faster imagined gait results in marked MLR activation during fMRI further implicating this region in locomotion (38).

Much of our understanding of the role of PPN in locomotion has been extrapolated from studies performed in non-human primates and rats. The rostral to caudal corticolimbic-ventral striatal-ventral pallidal-PPN-pontomedullary reticular nuclei-spinal cord pathway likely mediates various phases of locomotion including initiation, acceleration–deceleration, and termination of locomotion (31, 37, 39–41). The rostral to caudal locomotor pathway is modulated by parallel connections from the deep cerebellar and basal ganglia systems and integrated at the PPN, with prominent projections from the STN, GPi, and SN. Ventral pallidal outflow tracts to the PPN mediate spontaneous locomotor activity as evidenced by pharmacological manipulations performed in rat studies including (1) carbachol injection into unilateral PPN which led to reduced spontaneous locomotor activity and renormalization of enhanced locomotor activities seen with amphetamine injection into the nucleus accumbens and (2) procaine injection into the PPN suppressed the increased locomotor activity normally seen with hippocampal stimulation (42–44). MPTP intoxication in old, but not young monkeys induced balance and posture abnormalities; this was a significant finding because cholinergic neuronal loss in the PPN is present only in the older monkeys. Furthermore, direct bilateral PPN selective cholinergic neuron lesioning in monkeys (without MPTP treatment) causes axial rigidity and abnormalities of posture and gait without modifying overall global motor activity (38). Apomorphine administration improves the postural and gait abnormalities in monkeys previously treated with MPTP, but not in those receiving direct PPN cholinergic lesions (38, 45). The substantia nigra seems to play an important role in the initiation of locomotion and be potentially modulated by the PPN. The PPN likely modulates locomotor rhythmicity. Up to 70% of the neurons ventrally or dorsally adjacent to the PPN display burst firing which is correlated with cyclic frequencies of locomotion (46). By contrast, the majority of neurons in the PPN show tonic firing and are active during transitions of locomotion, activating or inactivating the rhythmic bursting neurons and firing transiently to initiate or terminate locomotion (46). Extrapolating from these animal studies, PPN circuitry may be involved in axial motor dysfunction (freezing/falling/swallowing) in the human.

Pathological and functional changes of the PPN have been observed in both animal models of PD and in patients with PD. A pathological study on eight patients with idiopathic PD revealed a 40% loss of large neurons within the PPN (39). Additionally, the severity of PPN neuronal loss was positively correlated with the severity of PD symptoms, and correlated to neuronal loss in the substantia nigra. Cholinergic neuron loss in the PPN was also greater in patients with PD who fell compared to similar PD patients who did not fall (39, 47). These observations add to our understanding of levodopa-resistant posture and gait deficits. Furthermore, the identification of cholinergic neurons specifically involved with control of gait and posture could improve targeting for DBS in this area. The close proximity of cholinergic and non-cholinergic regions within the PPN may result in current spread from one region to the other, therefore careful studies correlating exact electrode location and clinical effects are needed.

In PD, the increased inhibitory (GABAergic) activity from the GPi is thought to inhibit the PPN, and this has been shown in non-human primate models of PD revealing diminished PPN firing rates (48, 49). Further, stimulation or disinhibition of PPN via GABA antagonists has been shown to alleviate akinesia. However, these findings have not been universal and several groups have suggested that there is PPN overactivity in PD and that DBS benefits may be due to dampening PPN activity (23). In summary, PPN activity in PD is not clearly understood.

There is an ongoing debate as to where the PPN is located, and where groups have actually targeted the nucleus (e.g., PPN versus peri-peduncular)(50, 51). The mixed clinical outcomes in PPN DBS studies may therefore be a function of targeting variability. From a logistical standpoint, typical DBS fields of stimulation range between 2 and 3 mm and targeting errors range up to 3 mm. Computational modeling has shown that even small surgical targeting errors of 1 mm can lead to large decrements in activation of PPN (52). In addition, the PPN is anatomically and neurochemically heterogenous with subdivisions containing cholinergic, GABAergic, and glutamatergic neurons. With current stimulation parameters, current spread will necessarily affect multiple cell types. Due to these technical challenges, satisfactory PPN targeting may require further refinements in lead design. Some of these refinements may include PPN specific leads with small diameters, and with an increased number of electrical contacts and with a reduced intercontact distance, or alternatively with current steering technologies.

**Figure 2:**
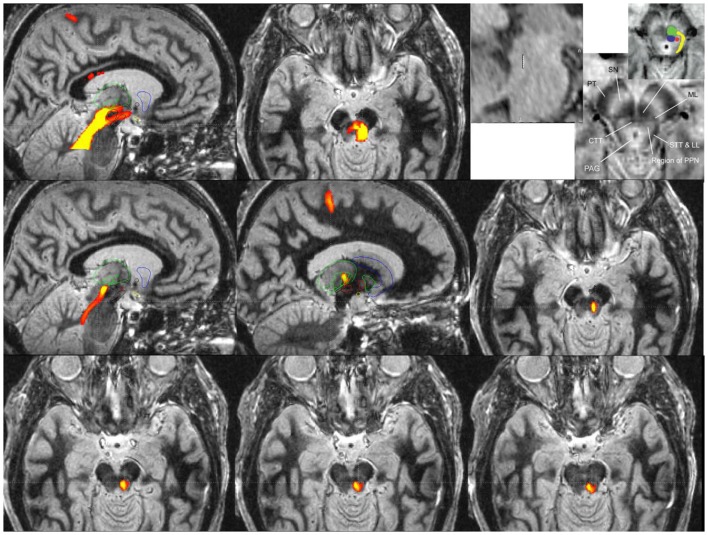
**Probabilistic diffusion tractography for surgical targeting and planning of a PPN DBS implantation guided by an fast gray matter acquisition T1 inversion recovery (FGATIR) MRI scan**.

Since microelectrode recording and plain MRI imaging cannot clearly delineate PPN borders, there has been a movement to exploit diffusion tension imaging (DTI) in order to identify tracts originating and terminating in the PPN. Indirect MRI based targeting can be used to define an approximate region for PPN DBS. One common targeting method has utilized the mean coordinates for the caudal and rostral edges of the PPN provided by Zrinzo et al. (see Figure 2). Direct MRI based targeting (i.e., T1 and FGATIR imaging can be used to reveal large contrast differences between gray and white matter) and to directly delineate a voxel corresponding to the midpoint of PPN. The midpoint voxel can be expanded in each direction creating a large cube (~6 × 6 × 6 mm^3^) to contain the general boundaries of PPN. Since the extent of the PPN is not well defined from anatomic imaging alone, diffusion tractography can be used to identify a more specific tract of interest for stimulation. Tractography from the PPN cube seed region can be used to reveal numerous subcortical connections including the thalamus, STN, SNr, and pallidum (53). Cortical connections can include the somatosensory cortex (S1), primary motor cortex (M1), supplementary motor areas, and premotor cortex (see Figure 2). Final refinement of the target can then be achieved by limiting tractography to the particular functional connection of interest. In order to identify the tract for lead placement, only fibers from the previously mentioned PPN cube (which pass through ventrolateral thalamus) that terminate in the motor cortex should be traced. Lead placement can be guided by selecting a trajectory that follows the tract from the region containing PPN to the lower trunk (e.g., legs) representation of motor cortex (specifically M1). Microelectrode recording passes can be also be used to differentiate PPN from adjacent structures with the data used in physiological research and potentially for guiding chronic stimulation settings. Though it is not yet feasible to clearly delineate the boundaries of PPN using microelectrode recordings and no unique signature activity has been identified, neuronal activity in the PPN differs by statistical measures of spontaneous activity from neighboring structures. Microelectrode studies of PPN in human patients have shown that the PPN has different spontaneous activity with a higher proportion of neurons with bursting discharge when compared with regions dorsal or ventral to PPN (19, 54, 55). In these studies, local field potentials recorded from the PPN showed greater power in the beta frequency range, potentially aiding in localization. Wide duration action potentials were thought to correspond with cholinergic neurons in PPN (54, 55). A combined approach utilizing tractography and microelectrode recording is one possible way investigators can try to narrow the target region for PPN DBS, but more research will be needed.

Nevertheless, the Toronto group’s surgical approach [using MRI-guided targeting and microelectrode recording (12)] has proven to be reasonably effective in targeting the PPN. Indeed, pathological analysis in two patients with progressive supranuclear palsy (PSP) who had undergone unilateral PPN DBS surgery has confirmed the position of the electrodes in the PPN region (56).

Probabilistic diffusion tractography maps in Figure 2 were created using in-house created software in conjunction with the FMRIB Software Library (FSL). FSL was used to determine the parameters for a two-fiber diffusion model at each voxel. This fiber model was then used with the FSL software to perform probabilistic tractography. This type of tractography allowed for a representation of the relative cross-sectional probability of a streamline passing through a voxel. A high contrast MRI scan, FGATIR (57), was used to help delineate the anatomic region around the PPN. A large region of interest around the region of the PPN that fully encompasses the PPN was used as a seed for tractography. If no mask is used to limit the tractography results, probabilistic tractography results may not be specific enough to be useful for surgical targeting (as shown in the top row of Figure 2). The top row images are (from left to right) a sagittal view of tracts from a large seed region including the PPN with fibers traveling from the PPN to various cortical and subcortical areas, an axial view of tracts in the PPN, and sagittal and axial views adapted with permission from Zrinzo et al. (58, 59) that show the location of the PPN identified from anatomy (using T1 and proton density weighted MRI). The top row highlights the older technique for DTI of the region, where the fiber pathway would be difficult to target because of specificity issues. The upper right image (top row) is the figure from Zrinzo et al. [figures adapted from Zrinzo et al. (59), permission for use of figures was obtained] (58, 59) and what has been shown red is the PPN, yellow is the lemniscal system, green is the superior cerebellar peduncles, and blue is the central tegmental tract.

A specific waypoint mask can be used to limit the results of probabilistic diffusion tractography to only include tracts that pass through a particular region of interest. For Figure 2 middle and bottom rows, a region of interest mask was created around the M1 primary motor cortex. We used this mask to limit the tractography results to only include the region of the PPN that has fiber connections to the primary motor cortex (M1). Middle row images (from left to right) show a medial sagittal slice of tracts from M1 to the region of the PPN, a lateral sagittal slice of tracts from M1 to the region of PPN (the tracts are going through VL (motor) thalamus, specifically Vim, Vop, and Voa), and an axial view of the tract from M1 to PPN. The right image of the second row shows correspondence with the location of PPN (top right panel) given by Zrinzo et al. (58, 59). The third bottom row shows more caudal cuts of PPN into the pons. The two bottom rows reveal the potential specificity of the technique for targeting with the DBS lead.

The refined PPN tract matches the same area that is [the red dot (PPN)] shown in the Zrinzo’s work, and it is felt to be likely the PPN proper. The top row images show the current and less specific methods to localize PPN and its tracts. The tracts likely include areas that are not PPN. The bottom rows are tracts from M1 to the region including PPN based on the University of Florida methodology. The idea of locating PPN should focus on subcortical connections and also those connections to motor cortex. Theoretically, a DBS lead (3389, 4 1.5 mm contacts with 1.5 mm spacing) can be placed into the tract to give the programmer in the clinic many options including the center of the PPN region itself (deepest lead), and three contacts along the PPN tract headed toward cortical and subcortical regions of interest, specifically the thalamus and ultimately the M1 region. However, specific leads designed for PPN DBS may be needed to achieve more consistent results. It is unknown whether this type of DTI imaging approach will ultimately improve targeting and outcomes; however, because of the uncertainty of the location of the PPN target, refinement techniques such as this will need to be further developed.

## Unilateral Versus Bilateral PPN DBS

Although the initial reports of PPN surgery in humans involved patients with advanced PD who had previously or concurrently undergone bilateral STN DBS, most previous clinical data were derived from unilateral PPN lesions or stimulation in normal or Parkinsonian animals. In a Parkinsonian monkey, unilateral PPN low frequency stimulation (LFS) improved generalized akinesia, whereas in Parkinsonian rats unilateral LFS of the PPN improved postural instability and contralateral limb use. These animal data suggest that unilateral PPN stimulation may thus have bilateral effects. To support these findings it has been shown in animals that the PPN has bilateral connections through a complex basal ganglia network (41). Moreover, bilateral PPN connections have been recently visualized in humans with PET studies (10).

The most recent clinical studies reporting effects of PPN DBS in PD patients have involved bilateral implantation with only few exceptions (10, 12, 16, 19, 21, 23, 26, 60, 61). Bilateral surgery has also been performed in a patient with primary progressive freezing gait disorder (13, 15). However, when comparing the French and Canadian studies, both with double blind measured changes in gait parameters (freezing) and balance as primary outcomes, unilateral and bilateral PPN stimulation achieved similar results in improving freezing at 1 year follow-up (12, 22). The group of Mazzone and coworkers has reported improvements in their technique with findings of similar benefit with unilateral PPN implantation (23).

Data from long-term observations are lacking, and the Toronto group has observed a progressive loss of the initial axial improvement (balance and freezing) after 3 years in two patients who had unilateral PPN DBS surgery. Interestingly, a new DBS surgery in the contralateral PPN restored the initial benefit in one patient and partially restored it in the other patient (personal communication Moro).

These findings raise several interesting questions that should be addressed in future studies:

Is it necessary to perform bilateral PPN DBS if a unilateral approach is safer and also potentially effective?
Should PPN DBS surgery be staged bilaterally or performed bilaterally and simultaneously in the same operating room sitting?
How much greater benefit, if any, is generally achieved with bilateral PPN DBS compared to unilateral PPN DBS? Furthermore, it is unknown whether there is lateralization of PPN function as has been described in limited studies of STN for PD (62, 63). fMRI studies of fast imagined walking in right handed normal individuals selectively activated the left PPN region suggesting a potential dominance of the left side for locomotor function. Additional activation has also been observed in the left SMA which is connected to the left PPN, and may thus be part of the dominant right leg representation. Comparing the effects of unilateral and bilateral stimulation in patients who have already undergone bilateral PPN electrode implantation along the lines of the Thevathasan et al. studies could potentially provide answers to these questions.

## Potential Approaches to PPN DBS Programing

Much of the data regarding DBS programing of the PPN have been extrapolated from studies performed in non-human primates and rats. When comparing the neuronal firing of the PPN with other basal ganglia nuclei (STN and GPi) in the Parkinsonian state, the PPN has revealed a much lower firing rate. In the normal rat, the PPN firing rate has been reported to fire at approximately 10 Hz, in comparison to the increased cell firing, irregularity and bursty pattern (18–20 Hz) observed in the Parkinsonian rat (64). PPN firing rates in PD patients have been described at approximately 14–25 Hz (19, 24, 54, 55).

Additionally, PPN stimulation with low and high frequency has shown different, and sometimes opposite effects when compared to STN stimulation. In normal non-human primates, PPN stimulation with low frequency (<30 Hz) induced tremor, whereas stimulation with high frequency (>100 Hz) decreased locomotor activity (65). In Parkinsonian non-human primates, PPN LFS at 5 Hz increased motor activity (54, 66–68) whereas PPN HFS resulted in akinesia (54, 66–68).

These non-human data have helped to direct the programing of PPN DBS in PD patients. Intraoperative data initially revealed improvement with LFS rather than HFS (24) but these results have not been consistently found during post-operative programing (9). Specifically, frequencies between 80 and 130 Hz have produced variable outcomes between groups ranging from no benefit (12, 24) to worsening (9) or to improvement (9) of gait scores.

Most groups have utilized PPN DBS at 25 Hz settings bilaterally (13, 18, 22, 69). However, no systematic investigation of low and high frequency stimulation was clearly performed in these studies. The Toronto group has systematically investigated different frequencies of stimulation (5, 20, 50, 70, and 130 Hz) in the PD patient with unilateral stimulation, and observed the most beneficial effects occurring around 50–70 Hz (12). The other parameters of stimulation (pulse width and voltage) did not seem to differ across studies. The reasons for these different frequency effects remain unclear.

Prior STN DBS may affect PPN function and the response to PPN DBS. Vitek and coworkers have recently observed that in primates with MPTP induced Parkinsonism treated with high frequency STN DBS there was a marked reduction in PPN activity (70). By contrast, in the initial human studies STN stimulation had only mild effects on PPN firing (24). The effects of STN stimulation on PPN activity have not fully been characterized, however, in patients with high frequency STN DBS that initially improved and subsequently developed a gait disorder, switching to low frequency (60 Hz) STN DBS may improve gait presumably through relieving the inhibitory input of the STN on the PPN. Indeed, Khan et al. reported that high frequency Zi stimulation worsened postural stability when combined with low frequency PPN DBS, and that the benefit was only achieved by reducing STN stimulation frequency to 60 Hz (25). Data from several groups have suggested that 60 Hz STN stimulation was similarly effective for ameliorating Parkinsonism, suggesting this strategy will not negatively impact symptoms primarily captured by STN DBS with perhaps the exception of tremor (71). Differing locations for stimulation in the PPN region and differing patient phenotypes might also contribute to the wide variability in stimulation parameters reported. Further studies correlating electrode position and effective stimulation parameters in patients with and without simultaneous STN DBS will be critical to help guide future DBS programing.

In the absence of more extensive studies, a rational approach to PPN DBS programing would include the use of a systematic manipulation of stimulation frequencies, starting with LFS and then progressively increasing stimulation up to 70–80 Hz if needed and perhaps higher in select cases.

There are both predictable and unpredictable side effects associated with DBS programing. The predictable side effects are based simply on the anatomical relationships of neighboring structures and have been well delineated by Lozano and colleagues (72). Less predictable are the non-motor side effects of PPN DBS, due to our limited understanding of the PPN in modulating these functions.

Deep brain stimulation for GPi and STN has the advantage that many of the symptomatic benefits can be approximated with macrostimulation in the OR. Bradykinesia, tremor, and rigidity are amenable to in person testing. Surrogate testing for postural instability or gait freezing are not currently amenable to macrostimulation in the OR. Imagined gait has been used in protocols to study the role of PPN in gait, but would require validation in clinical studies (38, 73). Identification of clinical outcome measures that can predict response to PPN DBS will be critical to move the field forward.

## The Importance of Non-Motor Functions of the PPN Region

Non-motor symptoms (NMS) of PD in many cases overshadow the motor symptoms (74, 75) and severely affect the quality of life for PD patients. NMS include autonomic dysfunction (gastrointestinal disorders, orthostatic hypotension, erectile dysfunction, sweating and urinary abnormalities), sleep disorders (restless legs syndrome, periodic limb movements of sleep, excessive daytime sleepiness, insomnia, REM sleep behavior disorder, and obstructive sleep apnea), cognitive dysfunction, mood disorders, and fatigue.

The non-motor effects of PPN DBS are less well characterized when compared to the traditional targets of the STN or GPi. This is not unexpected, since the non-motor functions of the PPN region are not well understood in humans and additionally NMS in PD have been historically much less emphasized. Finally, PPN DBS has been performed mainly to target motor and not non-motor disability. As PPN DBS moves forward, careful examination and tracking of changes in non-motor features will be important.

The PPN is thought to be involved in sleep, cognition, and in processing sensory and behavioral information (35). Deep brain areas involved in sleep have been studied mainly in animals. In cats, some cholinergic neurons in the PPN region have shown increased firing rates during rapid eye movement (REM) sleep (REM “on” neurons) (76). The Toronto group has also demonstrated that the presence of ponto-geniculo-occipital waves during REM sleep in humans can be recorded from an externalized PPN electrode (77).

The effects of bilateral PPN DBS on REM sleep were previously reported in one PD patient who underwent bilateral STN and PPN DBS surgery (78). Bilateral PPN DBS at low frequency (25 Hz) was associated with increased REM sleep (around 13%). In the same patient, bilateral STN DBS improved sleep efficiency by 80%, but did not have a major impact on REM sleep duration. The authors suggested that LFS of the PPN might modify the functional activity of cholinergic neurons, activating muscarinic receptors, and rebalancing REM sleep physiology through associated thalamic projections. Alternatively, locus coeruleus involvement in the stimulation field may also explain some of the findings. The Toronto group has analyzed the effects of unilateral PPN DBS on sleep in three PD patients and also in two patients with PSP who also underwent PPN DBS (60). Unilateral stimulation was associated with increased nocturnal REM sleep. This improvement occurred without regard to the frequency of PPN DBS, which was lower in PSP patients (5 and 30 Hz) compared to PD patients (70 Hz). REM sleep behavior disorder, present in two patients prior to DBS, was not modified by PPN stimulation. The results preliminarily suggest that PPN DBS promotes REM sleep by increasing output from the PPN (REM on) and by transsynaptically inhibiting the nuclei receiving GABAergic afferents in the ventrolateral periaqueductal gray and lateral pontine tegmentum (REM off) (79). These effects were observed in a small group of PSP patients, and we therefore cannot assume that these effects occur in PD. More recently, two PD patients in the Toronto cohort had improvement in alertness with bilateral LFS PPN DBS (10–25 Hz), whereas higher frequencies (80 Hz) induced non-REM sleep in one of the patients (80). The potential beneficial effects of PPN DBS on sleep and attention will require further investigation.

Experimental data from animals lend support to the notion that there is a role of PPN in cognition. Indeed, bilateral lesions of the PPN region impact attention, executive abilities, working memory and learning (35). Very little data are available in humans. Working memory tasks were studied in five PD patients with bilateral PPN and STN DBS implanted for at least 3 months and they were compared to a group of non-surgical PD patients, and also to another group of healthy subjects (81). No data were available prior to surgery. Although three surgical patients scored below the normal range on the neuropsychological battery, bilateral PPN stimulation at low frequency reduced PD patients’ response times on both the verbal and the visual-object tasks in when compared to the condition without stimulation. These results might support the hypothesis that PPN DBS facilitates the speeded processing of information in the context of working memory, and this may possibly occur by improving attention (81). It is also possible that that this result may have been mediated by activation of ascending cholinergic neurons to the intralaminar thalamic nuclei resulting in cortical activation. Indeed, some FDG and H_2_O^15^ PET studies with PPN DBS have demonstrated increased prefrontal cortical activation. More recently, data coming from Oxford in 11 PD patients with bilateral PPN DBS and 1 patient with unilateral PPN DBS revealed an improvement in the speed of reaction with LFS (20–35 Hz) (21). However, the authors suggested that this improved reaction time was related to an improved motor performance, rather than to augmentation of attention. Further investigation is needed to determine whether enhancement of attentional mechanisms may actually underlie the improvement in gait observed with PPN DBS.

In summary, it is unclear what underpins improvements in non-motor functions with PPN DBS. However, since there is some evidence that STN and GPi DBS might have a negative impact on some cognitive aspects in PD (3–5), PPN DBS could possibly be a safer target in the cognitive domain, but there is limited data to draw this conclusion.

## Patient Selection

Although we have pharmacologic therapies for levodopa-responsive symptoms, and GPi and STN DBS that effectively treat many patients with motor fluctuations, our current surgical therapies produce minimal benefits beyond the best ON-medication state, and do not address axial PD symptoms. As noted above, levodopa-refractory freezing and postural instability with falls remain an important and unaddressed source of morbidity and mortality and this is a place where PPN DBS, if shown effective, could fill an important vacuum in the field.

There have been important lessons learned in the past decade about the importance of carefully choosing the right candidate for PD (non-PPN) DBS, and most expert teams now use strict criteria, an interdisciplinary review, and teams carefully choose specific and relevant outcome variables. In general, patients who are chosen for STN and GPi DBS have a good response to levodopa for the target symptoms (with the exception of medication refractory motor fluctuations and dyskinesias), and the best candidates have little cognitive impairment.

Response to levodopa is predictive of the response to STN and GPi DBS. The predictive factors for response to PPN DBS are unknown, and must be carefully defined in future studies. Unfortunately, many of the published studies have small numbers of patients (see Table 1) and have not clearly defined inclusion or exclusion criteria. Most have been open-label, non-randomized, and have not utilized a battery of standardized outcome measures. Finally, it has not been clear that these studies have focused efforts on a therapy for ON medication axial dysfunction.

## Designing the Studies Needed to Move the Field Forward

Future studies must address these problems in study design in order to best determine the indications for and usefulness of PPN DBS. First, inclusion criteria must strictly define and assess for the presence of levodopa-refractory postural instability and FOG. Ideally, patients should be studied at the peak effect of a suprathreshold dose of levodopa. In general, these problems should persist even when the patient has peak dose dyskinesias. The methods for assessing postural instability and gait problems should be standardized, sensitive, and specific. Although the UPDRS is useful, it includes few measures of postural instability and gait and so does not describe many of complex features of these problems which would be more effectively measured with a combination of clinical rating scales and biomechanical measures. For example, the Tinetti Performance Oriented Mobility Assessment, a falls diary, FOG questionnaire, and walking speed might be used in combination with objective biomechanical measures of lower extremity function and center of mass oscillations to assess dynamic postural control when standing and during locomotion. All of the pre-operative measures should be completed post-operatively at selected time points ON and OFF stimulation and also assessed with and without the combined effects of anti-Parkinson medication. All measures should be performed in a double-blind fashion and given the uncertainty about the true benefit of PPN DBS, randomization of patients to immediate versus delayed surgery would allow for a more rigorous evaluation of its benefits. Long-term follow-up may be needed to assess the time course of improvement of PPN DBS.

As discussed above, the relative benefits of unilateral versus bilateral PPN DBS are unknown and it is possible that the additional benefit of bilateral stimulation is exceeded by the risk of implanting a second electrode. Alternatively, we might find that, as in STN and GPi DBS, there are significant additional and perhaps synergistic motor effects. Comparing the effects of unilateral and bilateral stimulation in patients who have already undergone bilateral PPN electrode implantation could potentially provide answers to these questions.

There is a fairly large population of patients who have previously undergone STN or GPi DBS, and now are presenting with late development of levodopa and stimulation resistant postural instability and gait problems. It is quite likely that they will respond differently to PPN DBS than patients who are DBS naïve. As a result, patients who present primarily with levodopa-refractory postural instability and gait problems without prior DBS may need to be studied in a separate cohort from with those with prior DBS. The potential for development of late complications in combination with the expected progression of the underlying disease in these patients will require that patients be tracked over several years (likely 5–10 years) to assess for the durability of the response to PPN DBS.

## Conclusion

The PPN DBS field needs a large trial that is adequately powered to detect a meaningful treatment effect. It is unlikely that the field will be able to adequately determine the effectiveness through the use of small pilot studies. It may be necessary to undertake a well-designed medium sized study to evaluate the variability in response to some of the primary outcome measures suggested above in order to allow sample size calculation for a large multicenter trial.

As discussed above, a number of additional investigations should be an integral part of any large clinical trial of PPN DBS. Correlating electrode position to clinical effects on motor and non-motor effects of surgery and stimulation will be extremely important to allow further refinement of the procedure, especially with the future advent of new electrode designs and closed loop systems incorporating responsive stimulation. Investigations to better determine the mechanism by which PPN DBS works will also be important in refining the technique. Based on the animal data, PPN DBS likely increases rather than inhibits PPN output and may reduce aberrant neuronal synchrony. The effects of stimulation on cognition, mood (apathy, depression, and anxiety), behavior (including impulse control disorders and DDS), autonomic function (urinary function, bowel control and the cardiovascular system), and sleep all should be concomitantly examined. Adverse effects should be scrupulously gathered as part of a large scale trial. Quality of life over the short and long term should be carefully assessed to determine the overall impact of the procedure on the patient’s life. Finally, one could imagine that FOG may possibly be approached by a closed loop approach using PPN DBS.

## Conflict of Interest Statement

The authors have no relevant financial disclosures to this review. Dr. Okun (the senior author) serves as a consultant for the National Parkinson Foundation, and has received research grants from NIH, NPF, the Michael J. Fox Foundation, the Parkinson Alliance, Smallwood Foundation, the Bachmann-Strauss Foundation, the Tourette Syndrome Association, and the UF Foundation. Dr. Okun has previously received honoraria, but in the past >48 months has received no support from industry. Dr. Okun has received royalties for publications with Demos, Manson, Amazon, Smashwords, and Cambridge (movement disorders books). Dr. Okun is an associate editor for New England Journal of Medicine and Journal Watch Neurology. Dr. Okun has participated in CME activities on movement disorders (in the last 36) months sponsored by PeerView, Prime, and by Vanderbilt University. The institution and not Dr. Okun receives grants from Medtronic and ANS/St. Jude, and the PI has no financial interest in these grants. Dr. Okun has participated as a site PI and/or co-I for several NIH, foundation, and industry sponsored trials over the years but has not received honoraria.
